# S3-2 Are hospital and community based physical activity program really effective in changing physical activity behaviour: a mixed method study

**DOI:** 10.1093/eurpub/ckad133.015

**Published:** 2023-09-11

**Authors:** Olivier Riquier, Anne Vuillemin, Yacoubou Omorou, Aurélie Van Hoye

**Affiliations:** Université de Lorraine, APEMAC, équipe MICS, F-54000 Nancy, France; Université Côte d’Azur, LAMHESS, France; Université de Lorraine, APEMAC, équipe MICS, F-54000 Nancy, France; Marie Curie Research Fellow, University of Limerick, Ireland

## Abstract

**Purpose:**

Based on the demonstrated benefits of physical activity (PA) practice for chronic health disease, PA resumption programs have come alive in France. However, their effectiveness has not been scientifically tested. The PERSISTE project (Promotion and sustainability of health enhancing PA) compare the effectiveness on PA practice and its determinants of an hospital led and a community led resumptions programs.

**Methods:**

A mixed-methods QUAN(qual) study assessed PA practice and its determinants, both quantitatively through the IPAQ questionnaire and qualitatively (practice, barriers, facilitators, challenges) during semi-structured interviews. Data were collected among 69 patients (34 hospital led and 35 community led) at three time point: enrolment in the program, end of the program and 3-5 months post program. Data analysis included multivariate data analysis for quantitative data and thematic coding for qualitative data. Data integration considered quantitative data on PA levels as drivers for qualitative data analysis.

**Results:**

Quantitative results have shown no difference of PA levels between the two programs at each measurement time point. Moreover, a significant difference was found on PA levels for both program between before and directly after the resumption program, but not between before and 3-5 months post program. Qualitative findings suggest key factors to consider to sustain PA practice, like the short duration not allowing behaviour change. Difference in challenges between programs have been found. A request for more supervised support in patient orientation after the hospital led program can be noticed. Organizational problems, such as unavailability of infrastructure or costs have been highlighted in the community program. Specific leverage arms were identified for each program, such as follow-up appointments after the hospital program or the practice offer in a very similar context for the community program.

**Conclusions:**

This study compared two resumption programs in different context and showed no significant difference on PA levels, but difference in intervention mechanisms supporting PA sustainability. Practical implications on strengths and weakness of each type of program, as well as challenges for both can be derived from this study, to enhance program quality.

**Funding:**

PERSISTE project is funded by the “Comité Départemental Olympique Sportif de Seine-Saint-Denis”.

